# Preconditioning Human Adipose-Derived Stromal Cells on Decellularized Adipose Tissue Scaffolds Within a Perfusion Bioreactor Modulates Cell Phenotype and Promotes a Pro-regenerative Host Response

**DOI:** 10.3389/fbioe.2021.642465

**Published:** 2021-03-18

**Authors:** Tim Tian Y. Han, John T. Walker, Aaron Grant, Gregory A. Dekaban, Lauren E. Flynn

**Affiliations:** ^1^Department of Anatomy & Cell Biology, Schulich School of Medicine & Dentistry, The University of Western Ontario, London, ON, Canada; ^2^Division of Plastic and Reconstructive Surgery, Schulich School of Medicine & Dentistry, The University of Western Ontario, London, ON, Canada; ^3^Molecular Medicine Research Laboratories, Robarts Research Institute, The University of Western Ontario, London, ON, Canada; ^4^Department of Microbiology & Immunology, Schulich School of Medicine & Dentistry, The University of Western Ontario, London, ON, Canada; ^5^Department of Chemical and Biochemical Engineering, Faculty of Engineering, The University of Western Ontario, London, ON, Canada; ^6^Bone and Joint Institute, The University of Western Ontario, London, ON, Canada

**Keywords:** bioreactors, decellularized adipose tissue, preconditioning, angiogenesis, immunomodulation, macrophage, adipose-derived stromal cell

## Abstract

Cell-based therapies involving the delivery of adipose-derived stromal cells (ASCs) on decellularized adipose tissue (DAT) scaffolds are a promising approach for soft tissue augmentation and reconstruction. Our lab has recently shown that culturing human ASCs on DAT scaffolds within a perfusion bioreactor prior to implantation can enhance their capacity to stimulate *in vivo* adipose tissue regeneration. Building from this previous work, the current study investigated the effects of bioreactor preconditioning on the ASC phenotype and secretory profile *in vitro*, as well as host cell recruitment following implantation in an athymic nude mouse model. Immunohistochemical analyses indicated that culturing within the bioreactor increased the percentage of ASCs co-expressing inducible nitric oxide synthase (iNOS) and arginase-1 (Arg-1), as well as tumor necrosis factor-alpha (TNF-α) and interleukin-10 (IL-10), within the peripheral regions of the DAT relative to statically cultured controls. In addition, bioreactor culture altered the expression levels of a range of immunomodulatory factors in the ASC-seeded DAT. *In vivo* testing revealed that culturing the ASCs on the DAT within the perfusion bioreactor prior to implantation enhanced the infiltration of host CD31^+^ endothelial cells and CD26^+^ cells into the DAT implants, but did not alter CD45^+^F4/80^+^CD68^+^ macrophage recruitment. However, a higher fraction of the CD45^+^ cell population expressed the pro-regenerative macrophage marker CD163 in the bioreactor group, which may have contributed to enhanced remodeling of the scaffolds into host-derived adipose tissue. Overall, the findings support that bioreactor preconditioning can augment the capacity of human ASCs to stimulate regeneration through paracrine-mediated mechanisms.

## Introduction

Tissue-engineering strategies represent a promising approach for the long-term augmentation and regeneration of damaged or deficient subcutaneous adipose tissue for applications in plastic and reconstructive surgery. Recent advancements in the field have focused on using a combined approach involving cell-seeded scaffolds as a means to provide both immediate volumetric augmentation and promote the stable regeneration of host-derived soft tissues (Choi et al., 2009; Brett et al., 2017). In particular, decellularized adipose tissue (DAT) has emerged as a promising pro-adipogenic platform for this application (Flynn, 2010; Mohiuddin et al., 2019). DAT scaffolds can be fabricated from human adipose tissue that is abundantly discarded as surgical waste, and have been shown to retain biochemical and biophysical properties that mimic the native tissue source, which may be favorable for adipose tissue regeneration (Haddad et al., 2016; Kuljanin et al., 2017).

While applying DAT as an off-the-shelf scaffold is appealing, studies have suggested that the rate and extent of adipose tissue regeneration can be enhanced by seeding the scaffolds with pro-regenerative progenitor cells (Young et al., 2014; Wang et al., 2013). This strategy may be critical for the larger volume augmentation that is required for many reconstructive applications in the clinic, as a lack of angiogenesis and host cell infiltration can result in implant failure. Previous work from our lab supports that seeding the DAT with adipose-derived stromal cells (ASCs) can enhance *in vivo* angiogenesis and host adipogenesis in immunocompetent mouse and rat models (Han et al., 2015; Robb et al., 2020). ASCs are a logical cell source for this application given their relative abundance and accessibility (Bourin et al., 2013), their high tolerance of ischemic conditions such as those immediately following implantation (Suga et al., 2010), as well as their enhanced adipogenic potential compared to other mesenchymal stromal cell (MSC) sources (Pizzute et al., 2015). Most clinical plastic surgery studies to date have focused on using the stromal vascular fraction (SVF) of adipose tissue to avoid the translational hurdles associated with the use of cultured ASC populations. However, through careful consideration of the cell culture microenvironment, it may be possible to design systems for cell expansion and preconditioning that would augment the capacity of the ASCs to stimulate regeneration, resulting in a more robust and predictable response that would justify the additional costs and regulatory hurdles involved.

In our previous work, the static seeding methods used resulted in a sparse and heterogeneous spatial distribution of ASCs on the DAT scaffolds, which may have restricted their capacity to stimulate regeneration. To address this limitation, we recently investigated the effects of culturing human ASCs on the 3-D DAT scaffolds used for cell delivery within a scaffold-based perfusion bioreactor system (Han and Flynn, 2020). Our findings demonstrated that dynamic culture under 2% O_2_ promoted human ASC expansion in the peripheral regions of the DAT. Further, culturing within the bioreactor under 2% O_2_ for 14 days prior to implantation significantly augmented blood vessel infiltration and host-derived adipose tissue formation within the DAT scaffolds in a subcutaneous implant model in athymic nude (*nu/nu*) mice in comparison to scaffolds cultured within the bioreactor under 20% O_2_, as well as statically cultured, freshly seeded, and unseeded controls (Han and Flynn, 2020). While the restrictions on the implant size and differences in the structure of the skin make it challenging to translate the findings of mouse models to predict future outcomes in humans, *in vivo* studies in immunocompromised mice remain a valuable tool for characterizing the effects of human ASCs in a complex physiological environment and for comparing the efficacy of varying ASC culture strategies or delivery platforms.

Although the delivery of a higher density of ASCs within the DAT scaffolds likely contributed to the enhanced adipose tissue regeneration observed in the 2% O_2_ bioreactor group, the dynamic culture conditions may have also preconditioned the ASCs to have a more pro-regenerative phenotype. A growing body of evidence supports that ASCs delivered within scaffolds primarily stimulate regeneration through transient paracrine-mediated effects, rather than through long-term engraftment and differentiation (Chazenbalk et al., 2011; Suga et al., 2014; Kang et al., 2014). More specifically, ASCs can secrete a diverse range of growth factors and cytokines that can promote the recruitment and/or modulate the response of host cells, including endothelial cells, adipogenic progenitors, and immune cell populations that can contribute to implant remodeling and *de novo* adipose tissue formation (Kapur and Katz, 2013). While the stimulatory effects of hypoxia on pro-angiogenic factor and cytokine secretion are well documented (Thangarajah et al., 2009; Hsiao et al., 2013), the effects of dynamic culture on MSC paracrine factor expression remain largely unexplored, with most bioreactor studies to date focused on characterizing the effects on proliferation and/or differentiation (Zhao and Ma, 2005; Alvarez-Barreto et al., 2011; Dos Santos et al., 2014; Yu et al., 2017).

Recognizing that dynamic culture may enhance the pro-regenerative capacity of the ASCs, we hypothesized that culturing the ASCs on the DAT scaffolds within the perfusion bioreactor would modulate their phenotype and paracrine function. Building from our previous work, human ASCs were cultured on DAT scaffolds under 2% O_2_ either within the perfusion bioreactor or statically within the culture inserts for 14 days. *In vitro* analyses were first performed to assess the effects of dynamic culturing on ASC phenotype and paracrine factor expression. Subsequently, the impact of bioreactor preconditioning on the pro-regenerative paracrine functionality of the ASC-seeded DAT scaffolds was explored by characterizing host cell recruitment at 1-, 4-, and 8-weeks post-implantation in the athymic nude mouse model.

## Materials and Methods

### Materials

Unless otherwise stated, all reagents were purchased from Sigma Aldrich Canada Ltd., and used as received (Oakville, Canada).

### Human Adipose Tissue Collection and Processing

Surgically discarded subcutaneous adipose tissue was collected with informed consent from female donors undergoing elective lipo-reduction surgeries at the University Hospital or St. Joseph’s Hospital in London, ON, Canada. Human Research Ethics Board approval for this study was obtained from Western University (HSREB# 105426). The fresh tissues were transported to the lab in sterile phosphate buffered saline (PBS) supplemented with 2% bovine serum albumin (BSA) on ice. Within 2 h of collection, the adipose tissue was processed for either ASC isolation or decellularization following established protocols (Flynn, 2010). The human ASCs were cultured on T75 flasks (Corning, Fisher Scientific, Ottawa, Canada) in proliferation medium (DMEM:Ham F12 (Wisent, St. Bruno, Canada) supplemented with 10% fetal bovine serum (FBS; Wisent, St. Bruno, Canada) and 100 U/mL penicillin and 0.1 mg/mL streptomycin (1% pen-strep; Thermo Fisher Scientific, Waltham, MA, United States).

To prepare the DAT scaffolds for cell culture, DAT samples were lyophilized and cut into individual scaffolds having a mass of 8 ± 1 mg, sized to fit within the cylindrical culture inserts used for both groups in this study (height 11 mm, inner diameter 5 mm) (Han and Flynn, 2020). The scaffolds were rehydrated in deionized water, decontaminated through repeated rinsing in 70% ethanol, and washed in sterile PBS. Prior to seeding, the scaffolds were equilibrated in fresh proliferation medium for 24 h.

### Scaffold Seeding and Culture

Adipose-derived stromal cells were seeded at passage 3 at a density of 1 × 10^6^ cells/scaffold as previously described (Han and Flynn, 2020), transferred into the culture inserts, and cultured in proliferation medium for 14 days either statically in 15 mL vented-cap conical tubes or dynamically within a customized perfusion bioreactor system (Tissue Growth Technologies, Instron) under a flow rate of 0.5 mL/min (Han and Flynn, 2020). Both the dynamic and static control samples were cultured within the cylindrical inserts (Han and Flynn, 2020) to ensure that the scaffold geometry and the surface area that was in direct contact with the media prior to perfusion were consistent between the groups. Hypoxic conditions (2% O_2_/93% N_2_/5% CO_2_, 37°C) were maintained for all cultures using a tri-gas incubator (ThermoFisher Forma Series II 3110).

### Immunohistochemical Assessment of the Effects of Bioreactor Culture on the DAT on Phenotypic Markers

Immunohistochemical analyses were performed to assess the expression of inducible nitric oxide synthase (iNOS), a marker associated with the immunomodulatory function of murine MSCs (Ren et al., 2008; Li et al., 2012; Maria et al., 2018), as well as arginase-1 (Arg-1), in the ASCs within the peripheral (<200 μm from the scaffold edge) or central (>200 μm from the scaffold edge) regions of the DAT scaffolds cultured either dynamically or statically for 14 days. In addition, triple staining was performed for Arg-1 with the immunomodulatory cytokines interleukin-10 (IL-10) and tumor necrosis factor-α (TNFα). The primary and secondary antibodies and dilutions are summarized in Table 1.

**TABLE 1:** Primary and secondary antibodies and dilutions used for immunostaining in the *in vitro* studies.

**Primary antibody**	**Primary Dilution**	**Secondary antibody**	**Secondary Dilution**
Arg-1 (Millipore ABS535)	1:100	Goat anti-chicken Alexa^®^ 488 (Abcam ab150169)	1:200
iNOS (Abcam ab15323)	1:100	Goat anti-rabbit Alexa^®^ 594 (Abcam ab150080)	1:200
TNF-α (Abcam ab6671)	1:200	Goat anti-rabbit Alexa^®^ 594 (Abcam ab150080)	1:400
IL-10 (R&D AF519)	1:50	Donkey anti-goat Alexa^®^ 680 (ThermoFisher A-21084)	1:100

For both analyses, scaffolds were fixed in 4% paraformaldehyde for 24 h at 4°C before being embedded in paraffin and sectioned (7 μm sections). The sections were de-paraffinized in an ethanol series and heat-mediated antigen retrieval was performed by incubating in Tris-EDTA buffer (10 mM tris base, 1 mM EDTA, 0.05% Tween-20, pH 9.0) at 95°C on a hot plate for 25 min. The sections were cooled for 25 min and then blocked with 5% BSA in PBS-T (0.1% Tween 20) for 1 h at room temperature before being incubated with the primary antibodies for iNOS in combination with Arg-1 or Arg-1 in combination with TNF-α and IL-10 at 4°C overnight. Next, the sections were washed with PBS and incubated with the secondary antibodies for 1 h at room temperature. Mouse spleen and liver were used as tissue positive controls, and no primary antibody controls were included in all trials.

Stained cross-sections were mounted in Fluoroshield Mounting Medium with DAPI (Abcam, Cambridge, MA, United States) and visualized with an EVOS^®^ FL Cell Imaging System (Thermo Fisher Scientific). Positively stained cells from five non-overlapping fields of view from both the central and peripheral regions were quantified manually in a blinded fashion using ImageJ Software in three non-adjacent cross-sections taken at least 100 μm apart from each scaffold. For the analyses, grayscale images for each color channel were used, and a brightness threshold of 50–255 was applied with a particle size >50 used to quantify DAPI^+^ cells. A total of 3 trials were performed with ASCs from different donors (*N* = 3).

### Quantitative Analysis of the Effects of Perfusion Bioreactor Culture on Immunomodulatory Factor Expression in Human ASCs Cultured on the DAT

A Human Magnetic Luminex^®^ Assay was performed to compare the protein expression levels of a range of pro-angiogenic and immunomodulatory factors between the static and dynamic groups at 14 days. TNF-α and IL-10 were selected based on their well-recognized counterbalancing roles in regulating the inflammatory response, as well as to compare with the IHC findings. Hepatocyte growth factor (HGF) was chosen as a pleiotropic factor that is highly expressed by ASCs, which can have both pro-angiogenic and anti-inflammatory effects (Cai et al., 2007; Ceccarelli et al., 2020). Chemokine C-X-C motif 10 (CXCL-10) was selected as a marker associated with inflammation in adipose tissue (Kochumon et al., 2020), as well as macrophage recruitment in other tissues (Petrovic-Djergovic et al., 2015). Finally, C-X-C motif ligand 2 (CXCL-2) was chosen as a pro-inflammatory chemokine that has been shown to be upregulated during adipogenic differentiation (Kusuyama et al., 2016; Siebert et al., 2016), and interleukin-6 (IL-6) was included as an additional pro-inflammatory adipokine that can modulate both lipid metabolism and macrophage polarization within adipose tissue (Trujillo et al., 2004; Braune et al., 2017).

For this assay, three replicate scaffolds from each group (*n* = 3) were analyzed per ASC donor, and a total of five trials were performed with different ASC donors (*N* = 5). In preparation for the Luminex^®^ assay, the individual scaffolds were frozen in liquid nitrogen, crushed with a mortar and pestle, and resuspended in a lysis buffer (50 mM Tris-HCl, 100 mM NaCl, 10% glycerol, 1% Triton X-100, pH 7.4). The samples were then briefly sonicated with an ultrasonic dismembrator (ThermoFisher Model 100) and centrifuged at 13,000 × *g* for 10 min at 4°C. The supernatant from each sample was then analyzed with a Human Magnetic Luminex^®^ Assay (R&D Systems) using a MAGPIX^®^ System (Millipore), in accordance with the manufacturer’s protocols. Protein concentrations were determined based on comparison to standard curves and normalized to the double stranded DNA (dsDNA) content measured in each sample using a PicoGreen^®^ dsDNA Assay (Han and Flynn, 2020), following the manufacturer’s instructions.

### Subcutaneous Implantation Surgeries

*In vivo* studies were performed to compare the effects of culturing the ASCs on the DAT scaffolds for 14 days under static or dynamic conditions on the host cell response. All animal studies followed Canadian Council on Animal Care (CCAC) guidelines and the protocols were reviewed and approved by the Western University Animal Care Committee (Protocol #2015-049). Female athymic nude mice (Nu-*Foxn1*^*nu*^) (Charles River Laboratories, Sherbrooke, Canada) of 10–13 weeks of age were used for this study (*N* = 6 mice per scaffold group/timepoint). Subcutaneous implantation surgeries were performed following established protocols (Han and Flynn, 2020). At 1, 4, and 8 weeks, the mice were sacrificed by CO_2_ overdose and the scaffolds were excised within their surrounding tissues. The samples were fixed in 4% paraformaldehyde at 4°C overnight before being embedded in paraffin and sectioned (7 μm) for immunohistochemical analyses.

### Immunohistochemical Analysis of Host Cell Recruitment

Immunostaining was performed to assess host cell infiltration into the scaffolds at 1, 4, and 8 weeks. More specifically, CD31 staining was performed to examine host endothelial cell recruitment. In addition, co-staining was performed for CD26, a marker that has been associated with highly proliferative multipotent progenitors that give rise to preadipocytes in murine subcutaneous adipose tissue (Merrick et al., 2019), along with the human cell marker Ku80 (Allard et al., 2014), to distinguish the host-derived CD26^+^ population. Finally, host immune cell recruitment was characterized through triple staining for the pan-leukocyte marker CD45, the murine macrophage marker F4/80, and the phagocytic macrophage marker CD68. The phenotype of the infiltrating macrophages was also probed by co-staining for CD45 in combination with the pro-regenerative macrophage marker CD163. For each explanted scaffold that was analyzed (*N* = 6 for CD31, CD26 and CD163 analyses, *N* = 4 for macrophage recruitment), three non-adjacent cross-sections at least 100 μm apart were assessed. Immunohistochemical staining and imaging were performed following the methods described above and using the antibodies summarized in Table 2. Quantification was performed within 10 randomly selected and non-overlapping, non-adjacent fields of view in each section as previously described.

**TABLE 2:** Primary and secondary antibodies and dilutions used for immunostaining in the *in vivo* study.

**Primary antibody**	**Primary Dilution**	**Secondary antibody**	**Secondary Dilution**
CD31 (Abcam ab28364)	1:100	Goat anti-rabbit Alexa^®^ 594 (Abcam ab150080)	1:200
Ku80 (Cell Signaling 2180)	1:200	Goat anti-rabbit Alexa^®^ 594 (Abcam ab150080)	1:400
CD26 (R&D AF954)	1:50	Donkey anti-goat Alexa^®^ 680 (ThermoFisher A-21084)	1:100
CD45 (R&D AF114)	1:100	Donkey anti-goat Alexa^®^ 680 (ThermoFisher A-21084)	1:200
CD68 (BioRad MCA1957)	1:100	Goat anti-rat Alexa^®^ 488 (ThermoFisher A-11006)	1:200
F4/80 (Abcam ab111101)	1:50	Goat anti-rabbit Alexa^®^ 594 (Abcam ab150080)	1:100
CD163 (Abcam ab182422)	1:200	Goat anti-rabbit Alexa^®^ 594 (Abcam ab150080)	1:400

### Statistical Methods

Statistical analyses were performed using linear mixed effects models and multiple comparisons were corrected as described by Hothorn et al. (2008). Statistical analyses were performed with R statistics software (R Core Team, 2017) using the “lme4” package for linear and non-linear mixed effects models (Bates et al., 2015), and the “multcomp” package for simultaneous inference in general parametric models (Hothorn et al., 2008). For *in vitro* immunofluorescence studies, sample location (peripheral/central) and treatment (static/dynamic) were included as fixed effects, and ASC donor was included as a random effect. For the Luminex^®^ assay, treatment was included as a fixed effect and ASC donor was included as a random effect. For analyses of *in vivo* samples, treatment and time were included as fixed effects, and ASC donor and mouse were included as random effects. Corrected *p*-values <0.05 were considered to be statistically significant. Graphs were produced using GraphPad Prism version 6 (GraphPad, La Jolla, CA, United States). Error bars represent the standard deviation.

## Results

### Dynamic Culture Increased the Percentage of ASCs Expressing iNOS Within the Peripheral Regions of the DAT Scaffolds

**FIGURE 1:**
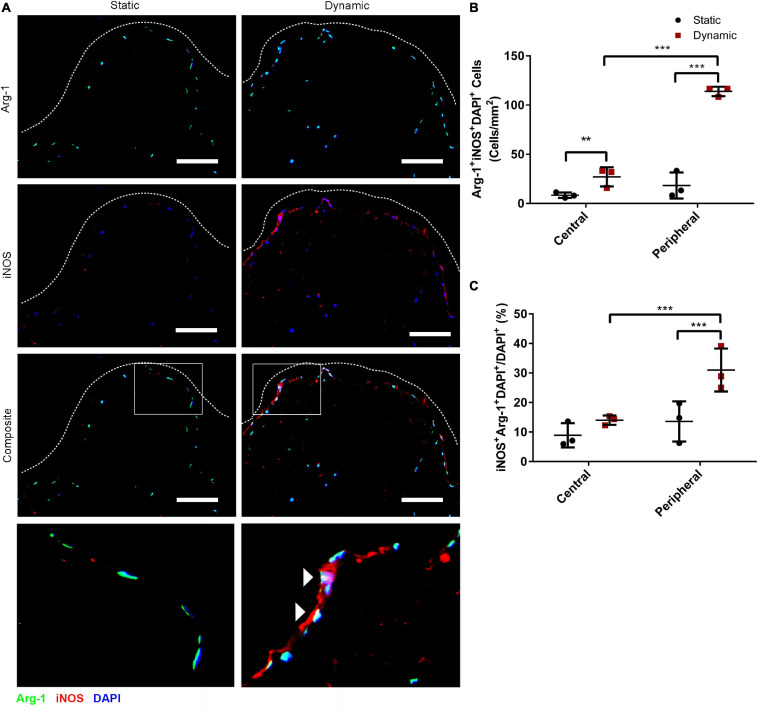
Dynamic culture within the perfusion bioreactor for 14 days increased the percentage of human ASCs co-expressing iNOS and Arg-1 within the peripheral region of the DAT scaffolds. **(A)** Representative immunostaining for Arg-1 (green) and iNOS (red) with DAPI (blue) for the static and dynamic groups at 14 days. Dashed lines indicate the edge of the DAT scaffolds. Scale bars represent 100 μm. Boxed regions in the composite images are shown at higher magnification below. White arrowheads highlight Arg-1^+^iNOS^+^DAPI^+^ cells. **(B)** The density of iNOS^+^Arg-1^+^DAPI^+^ cells and **(C)** the percentage of iNOS^+^Arg-1^+^DAPI^+^ cells relative to the total DAPI^+^ cell population in the central (>200 μm from scaffold edge) and peripheral (<200 μm from scaffold edge) regions of the DAT scaffolds cultured statically or dynamically. **p < 0.01, ***p < 0.001.

Immunostaining was performed to assess the expression of iNOS and Arg-1 in the ASCs that were cultured statically or dynamically on the DAT scaffolds for 14 days. A high density of iNOS^+^ cells was visualized along the peripheral edges of the scaffolds in the dynamic group, while Arg-1^+^ cells were distributed throughout the scaffolds in both groups (Figure 1A). Consistent with our previous findings that dynamic culture within the bioreactor under 2% O_2_ promoted ASC expansion within the periphery of the DAT (Han and Flynn, 2020), DAPI quantification confirmed that the ASC density was significantly higher in the peripheral region (<200 μm from scaffold edge) of the dynamic group (562 ± 116 cells/mm^2^), as compared to the central region (>200 μm from scaffold edge) of the dynamic group (213 ± 54 cells/mm^2^) and the peripheral region of the static group (284 ± 80 cells/mm^2^) (Supplementary Figure 1).

Quantification of iNOS^+^Arg-1^+^DAPI^+^ cells in the peripheral and central regions of the scaffolds was performed. A significantly higher density of iNOS^+^Arg 1^+^DAPI^+^ cells was observed in the peripheral region of the dynamic group (114 ± 5 cells/mm^2^) as compared to the peripheral region of the static group (18 ± 13 cells/mm^2^) and the central region of the dynamic group (27 ± 10 cells/mm^2^) (Figure 1B). The data was also analyzed as a percentage to account for differences in the total cell density in the two regions for each of the groups, and assess whether there was significant upregulation in the relative levels of positive cells with media perfusion. Analysis of the iNOS^+^Arg-1^+^DAPI^+^ cell population relative to the total DAPI^+^ cell population confirmed that a significantly higher percentage of the ASCs co-expressed iNOS and Arg-1 within the peripheral region in the dynamic group (30.9 ± 7.3%) as compared to the peripheral region of the static group (13.6 ± 6.8%) and the central region of the dynamic group (14.0 ± 1.6%) (Figure 1C), suggesting that culturing within the perfusion bioreactor altered the phenotype of the ASCs within the scaffold periphery.

Further probing the numbers of cells expressing the individual markers, the density of iNOS^+^DAPI^+^ cells was significantly higher in the peripheral region of the dynamic group (122 ± 2 cells/mm^2^) as compared to the peripheral region of the static group (22 ± 16 cells/mm^2^) and the central region of the dynamic group (32 ± 8 cells/mm^2^) (Supplementary Figure 2A). When assessed relative to the total DAPI^+^ cell population, the percentage of iNOS^+^DAPI^+^ cells was highest in the peripheral region of the dynamic group (33.2 ± 7.5%), and significantly greater than both the central region of the dynamic group (16.6 ± 0.8%) and the peripheral region of the static group (14.5 ± 7.4%) (Supplementary Figure 2B). A significantly higher density of Arg-1^+^DAPI^+^ cells was also observed in the peripheral region of the dynamic group (306 ± 68 cells/mm^2^) as compared to the peripheral region of the static group (117 ± 38 cells/mm^2^) and the central region of the dynamic group (146 ± 51 cells/mm^2^) (Supplementary Figure 2C). However, when evaluated relative to the total DAPI^+^ population, there were no significant differences between the regions or groups, with a high percentage (>70%) of the ASCs expressing Arg-1 under all conditions (Supplementary Figure 2D).

### Dynamic Culture Increased the Density of ASCs Expressing TNF-α and IL-10 in the DAT Scaffolds at 14 Days

**FIGURE 2:**
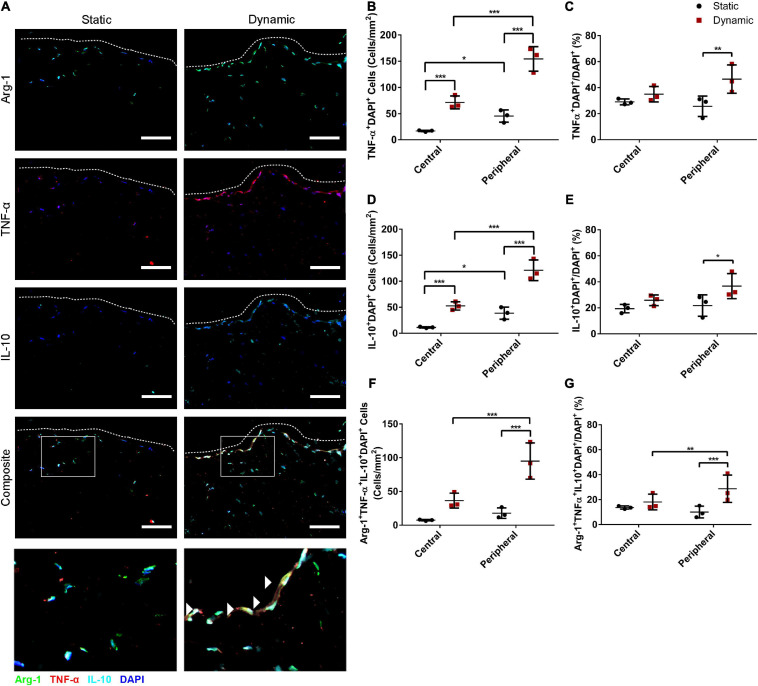
Dynamic culture within the perfusion bioreactor for 14 days increased the percentage of human ASCs expressing TNF-α^+^ and IL-10^+^ in the peripheral region of the DAT scaffolds. **(A)** Representative immunostaining showing Arg-1 (green), TNF-α (red), and IL-10 (cyan) expression with DAPI counterstaining (blue) for the static and dynamic groups. Dashed lines indicate the edge of the DAT scaffolds. Scale bars represent 100 μm. Boxed regions in the composite images are shown at higher magnification below. White arrowheads highlight Arg-1^+^TNF-α^+^ IL-10^+^DAPI^+^ cells. In the central (>200 μm from scaffold edge) and peripheral (<200 μm from scaffold edge) regions of the DAT scaffolds in the static and dynamic groups: **(B)** The TNF-α^+^DAPI^+^ cell density and **(C)** Percentage of cells expressing TNF-α. **(D)** The IL-10^+^DAPI^+^ cell density and **(E)** Percentage of cells expressing IL-10. **(F)** The Arg-1^+^TNF-α^+^IL-10^+^DAPI^+^ cell density and **(G)** Percentage of cells co-expressing Arg-1, TNF-α and IL-10. *p < 0.05, **p < 0.01, ***p < 0.001.

Immunostaining was performed for Arg-1 in combination with TNF-α and IL-10 to determine whether dynamic culture modulated the abundance and distribution of ASCs expressing these cytokines. High densities of Arg-1^+^, TNF-α^+^, and IL-10^+^ cells were observed within the peripheral regions of the DAT scaffolds in the dynamic group (Figure 2A). Quantification of the TNF-α^+^DAPI^+^ cells in the peripheral (<200 μm from scaffold edge) and central (>200 μm from scaffold edge) scaffold regions revealed a significantly greater density of positive cells in the peripheral region of the dynamic group (154 ± 23 cells/mm^2^) as compared to the peripheral region of the static group (46 ± 11 cells/mm^2^) and the central region of the dynamic group (72 ± 12 cells/mm^2^) (Figure 2B). In addition, the TNF-α^+^DAPI^+^ cell density was significantly greater in the central region of the dynamic group as compared to the central region of the static group (17 ± 1 cells/mm^2^). When expressed as a percentage of the total DAPI^+^ cell population, a significantly higher percentage of cells were TNF-α^+^ in the peripheral region of the dynamic group (46.6 ± 10.9%) as compared to the static group (25.7 ± 7.9%) (Figure 2C).

Similarly, there were significantly more IL-10^+^ cells in the peripheral region of the dynamic group (121 ± 20 cells/mm^2^) as compared to the peripheral region of the static group (39 ± 12 cells/mm^2^) and the central region of the dynamic group (52 ± 8 cells/mm^2^), which was significantly greater than the central region of the static group (11 ± 1 cells/mm^2^) (Figure 2D). When analyzed as a percentage, a significantly higher percentage of cells in the peripheral region were IL-10^+^ in the dynamic group (36.7 ± 9.7%) as compared to the static group (21.8 ± 8.2%) (Figure 2E).

Quantification also indicated that the Arg-1^+^TNF-α^+^IL-10^+^DAPI^+^ cell density was significantly higher in the peripheral region of the dynamic group (95 ± 27 cells/mm^2^) as compared to the peripheral region of the static group (18 ± 8 cells/mm^2^) and the central region of the dynamic group (36 ± 11 cells/mm^2^) (Figure 2F). Furthermore, a significantly higher percentage of the DAPI^+^ cells were TNF-α^+^IL-10^+^ (29.8 ± 11.3%) (Supplementary Figure 3) and Arg-1^+^TNF-α^+^IL-10^+^ (28.7 ± 10.9%) (Figure 2G) in the peripheral regions of the DAT scaffolds in the dynamic group as compared to the static group (11.2 ± 6.1% and 10.0 ± 4.7%, respectively). Taken together, these findings suggest that culturing in the perfusion bioreactor increased the fraction of ASCs that were expressing both TNF-α and IL-10 in the periphery of the DAT, and that the majority of these cells were also Arg-1^+^.

### Dynamic Culture Altered the Paracrine Factor Expression Profile of the ASCs in the DAT Scaffolds

**FIGURE 3:**
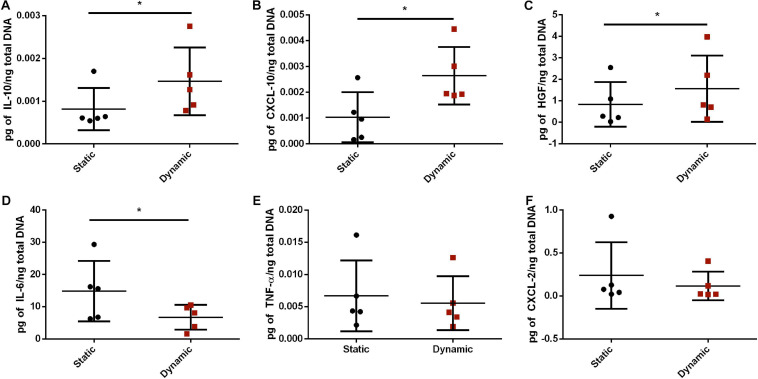
Dynamic culture within the perfusion bioreactor altered the paracrine factor expression profile of the human ASCs cultured on the DAT scaffolds for 14 days. Expression of **(A)** IL-10, **(B)** CXCL-10, **(C)** HGF, **(D)** IL-6, **(E)** TNF-α, and **(F)** CXCL-2 as measured by Human Magnetic Luminex^®^ Assay normalized to total dsDNA content. *p < 0.05.

To further probe whether bioreactor preconditioning altered the expression levels of some key immunomodulatory factors expressed within adipose tissue, a custom Luminex^®^ assay was performed. Protein expression levels of IL-10, CXCL-10, HGF, IL-6, TNF-α, and CXCL-2 were assessed in lysates prepared from the ASC-seeded DAT in the static and dynamic groups after 14 days of culture. After normalizing to total dsDNA content, the protein expression levels of IL-10, CXCL-10, and HGF were significantly higher, and IL-6 expression was significantly lower, in the dynamic group as compared to the static group (Figure 3). No significant differences were observed between the groups in the protein expression levels of TNF-α or CXCL-2.

### Dynamic Culture of ASCs on the DAT Enhanced CD31^+^ Endothelial Cell Recruitment Into the Scaffolds Following Implantation in the *nu/nu* Mouse Model

**FIGURE 4:**
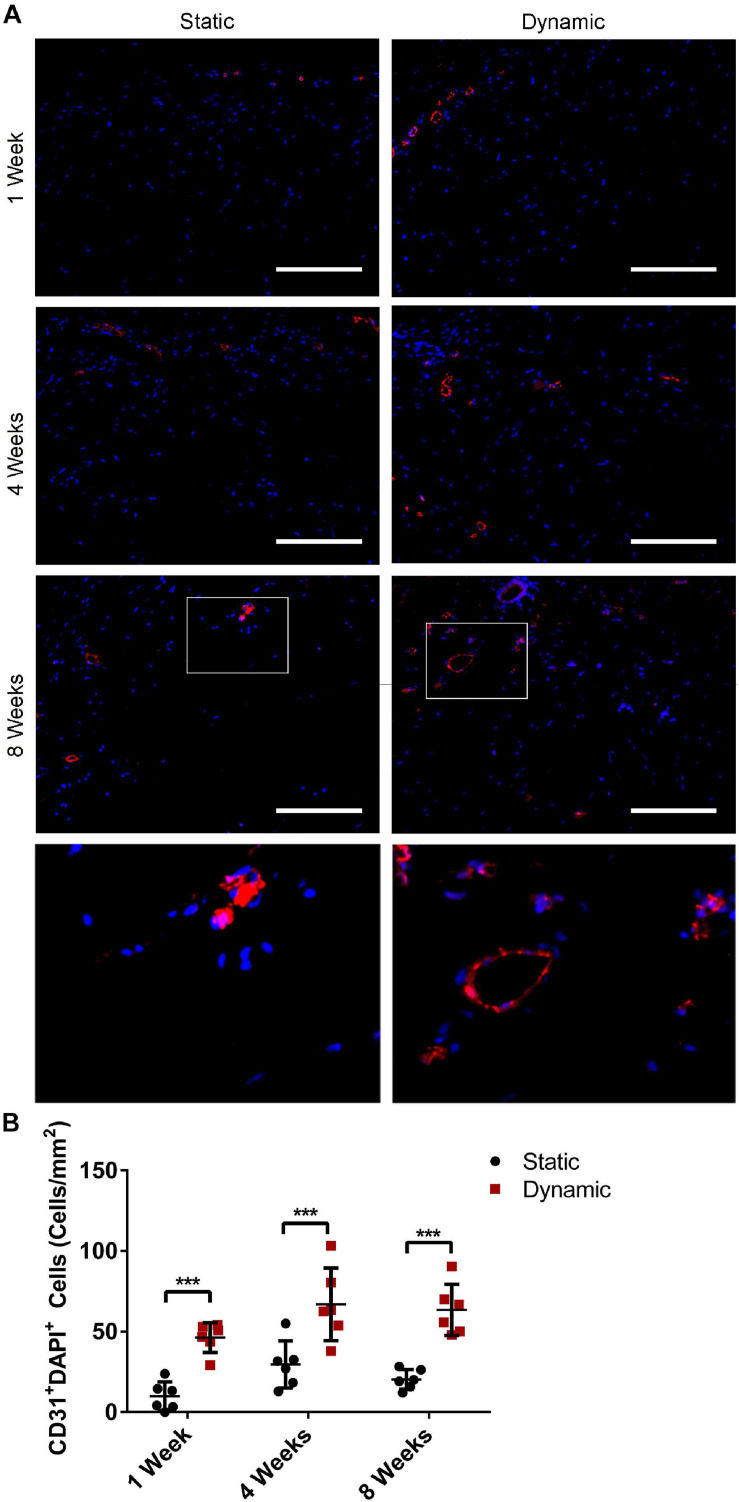
Dynamic culture of ASCs on the DAT scaffolds enhanced CD31^+^ cell recruitment following implantation in the *nu/nu* mouse model. **(A)** Representative immunostaining showing CD31 (red) with DAPI counterstaining (blue) within the DAT implants for both the static and dynamic groups at 1-, 4- and 8-weeks post-implantation. Scale bars represent 100 μm. Boxed regions in the 8-week images are shown at higher magnification below. **(B)** CD31^+^DAPI^+^ endothelial cell density in the implants. ***p < 0.001.

Following *in vitro* characterization, *in vivo* studies were performed to probe how bioreactor preconditioning modulated the capacity of the ASC-seeded DAT to stimulate the recruitment of a range of host cell populations at 1-, 4-, and 8-weeks post-implantation, which may have contributed to the markedly enhanced adipose tissue regeneration previously reported in this group (Supplementary Figure 4; Han and Flynn, 2020). Given the importance of angiogenesis in adipose tissue regeneration (Laschke et al., 2006), the initial characterization focused on assessing the presence and distribution of CD31^+^ endothelial cells within the implants (Figure 4A). Quantification of the staining confirmed that the CD31^+^ cell density was significantly greater in the dynamic group as compared to the static group at 1- (46 ± 9 cells/mm^2^ versus 10 ± 9 cells/mm^2^), 4- (67 ± 23 cells/mm^2^ versus 30 ± 15 cells/mm^2^), and 8-weeks (63 ± 16 cells/mm^2^ versus 20 ± 6 cells/mm^2^) post-implantation (Figure 4B), supporting that bioreactor preconditioning enhanced angiogenesis within the implants.

### Dynamic Culture of ASCs on the DAT Scaffolds Enhanced the Recruitment of CD26^+^ Host Cells at 1-Week Post-implantation in the *nu/nu* Mouse Model

**FIGURE 5:**
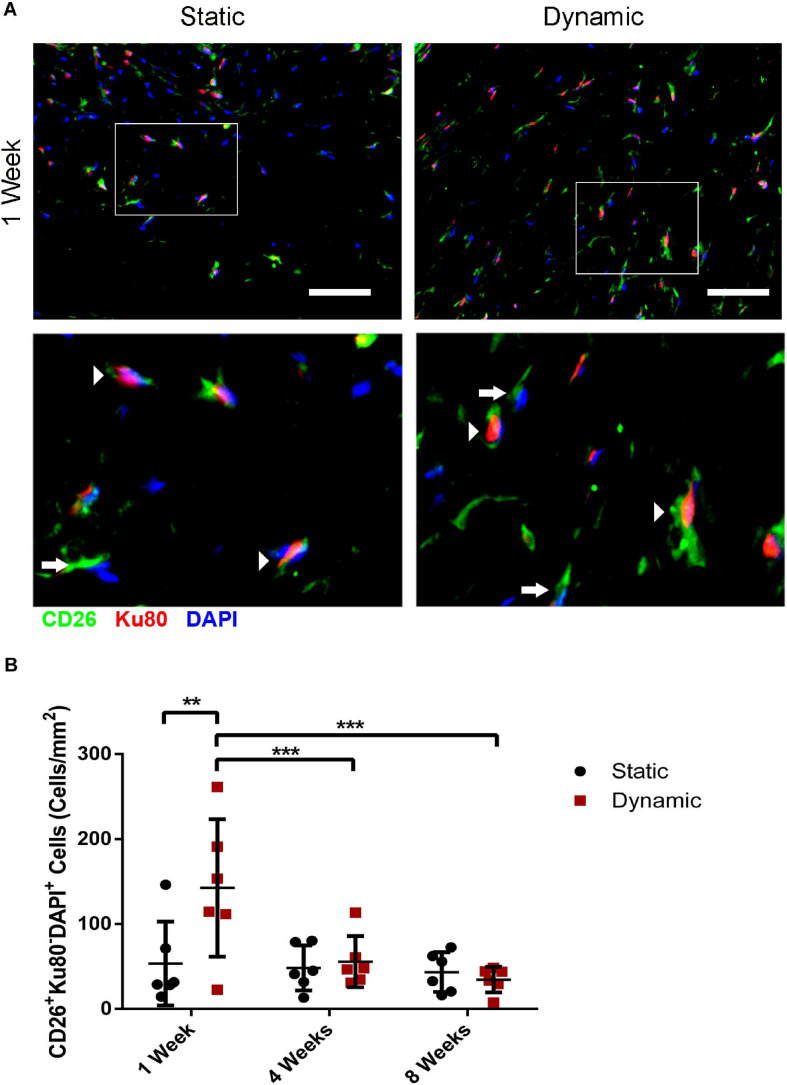
Dynamic culture of human ASCs on the DAT scaffolds enhanced the recruitment of CD26^+^ host cells at 1-week post-implantation in the *nu/nu* mouse model. **(A)** Representative immunostaining of CD26 (green) in combination with human-specific Ku80 (red), with DAPI counterstaining (blue) at 1-week post-implantation. Scale bars represent 50 μm. Boxed regions are shown at higher magnification below. Arrows indicate CD26^+^Ku80^–^ DAPI^+^ mouse cells and arrowheads highlight CD26^+^Ku80^+^DAPI^+^ human cells. **(B)** CD26^+^ host cell (CD26^+^Ku80^–^ DAPI^+^) density within the DAT implants at 1, 4, and 8 weeks. **p < 0.01, ***p < 0.001.

Co-staining was performed for CD26 and for human-specific Ku80 (Figure 5A and Supplementary Figure 5) to probe host CD26^+^ cell infiltration into the implants at 1, 4, and 8 weeks post-implantation, as a potential marker of multipotent progenitor cells that can give rise to preadipocytes and adipocytes (Merrick et al., 2019). Quantification of the CD26^+^Ku80^–^DAPI^+^ cell population indicated that there was a significantly higher density of CD26^+^ host cells in the DAT implants within the dynamic group at 1-week post-implantation (142 ± 81 cells/mm^2^) as compared to the static group (53 ± 49 cells/mm^2^), as well as the dynamic group at both 4- (56 ± 30 cells/mm^2^) and 8-weeks (35 ± 15 cells/mm^2^) post-implantation (Figure 5B).

Quantification of the Ku80^+^DAPI^+^ human ASC population was also performed. The Ku80^+^DAPI^+^ cell density was significantly higher in the dynamic group relative to the static group at 1-week post-implantation (196 ± 44 cells/mm^2^ versus 69 ± 18 cells/mm^2^), as well as the dynamic group at both 4- (81 ± 43 cells/mm^2^) and 8-weeks (62 ± 76 cells/mm^2^) post-implantation (Supplementary Figure 6). No significant differences were observed in the Ku80^+^DAPI^+^ cell density between the static and dynamic groups at 4 or 8 weeks. However, there was substantial variability in the dynamic group at 8 weeks, with fewer human cells present in the implants that had undergone more substantial remodeling into adipose tissue.

### Dynamic Culture of ASCs on the DAT Scaffolds Did Not Alter Host Macrophage Recruitment Following Implantation in the *nu/nu* Mouse Model

**FIGURE 6:**
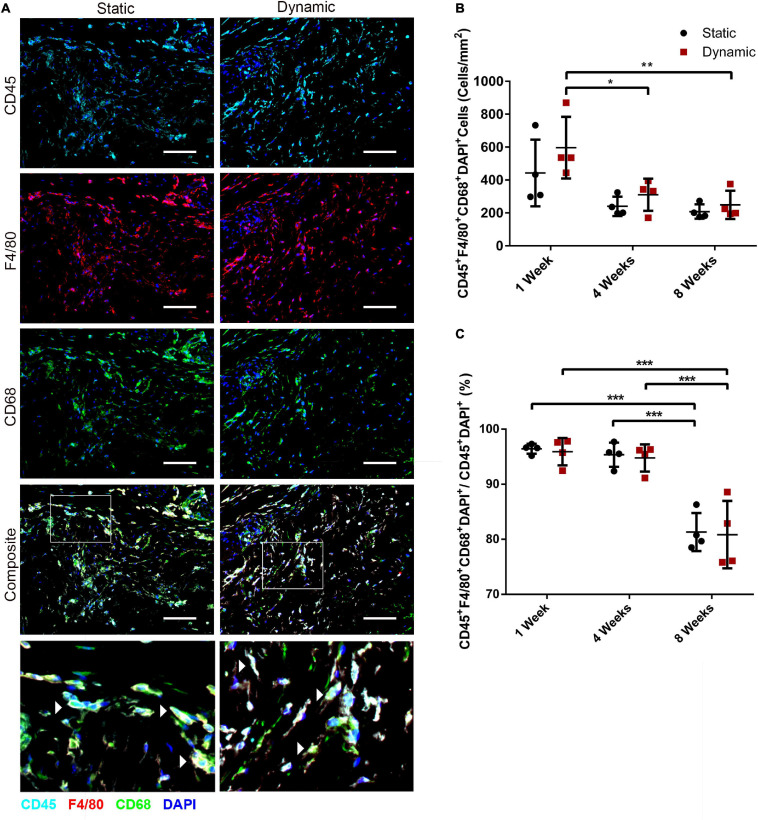
The recruitment of CD45^+^F4/80^+^CD68^+^ macrophages was similar in the ASC-seeded DAT scaffolds that were cultured dynamically and statically for 14 days prior to implantation in the *nu/nu* mouse model. **(A)** Representative images showing CD45 (cyan), F4/80 (red), and CD68 (green) expression with DAPI counterstaining (blue) at 1-week post-implantation. Scale bars represent 100 μm. Boxed regions in the composite images are shown at higher magnification below. White arrowheads highlight CD45^+^F4/80^+^CD68^+^DAPI^+^ cells. **(B)** CD45^+^F4/80^+^CD68^+^DAPI^+^ cell density in the static and dynamic groups. **(C)** The percentage of CD45^+^F4/80^+^CD68^+^DAPI^+^ cells relative to the total CD45^+^DAPI^+^ cell population at 1, 4, and 8 weeks. *p < 0.05, **p < 0.01, ***p < 0.001.

To probe host macrophage infiltration into the DAT implants over time, immunostaining was performed for mouse CD45 as a pan-leukocyte marker in combination with F4/80 as a marker of mature mouse macrophages, and CD68 as a marker of phagocytic macrophages. Qualitatively, high densities of CD45^+^, F4/80^+^ and CD68^+^ cells were observed within the DAT implants in both groups at 1- (Figure 6A), 4- (Supplementary Figure 7), and 8-weeks (Supplementary Figure 8) post-implantation. Quantification of the CD45^+^F4/80^+^CD68^+^DAPI^+^ cells indicated there was a significantly higher density in the dynamic group at 1-week post-implantation (596 ± 187 cells/mm^2^) as compared to both 4- (310 ± 97 cells/mm^2^) and 8-weeks (249 ± 86 cells/mm^2^) (Figure 6B). However, no significant differences were observed between the dynamic and static groups at any of the timepoints.

Analysis of the percentage of CD45^+^ cells that co-expressed both F4/80 and CD68 within the total CD45^+^DAPI^+^ cell population in the DAT implants indicated that the majority (∼95%) of the CD45^+^ cells were F4/80^+^CD68^+^ at 1 and 4 weeks, but a significant decline in this subpopulation was observed within both groups at 8 weeks, with (80.8 ± 6.1%) and (81.3 ± 3.5%) of the CD45^+^ population co-expressing F4/80^+^CD68^+^ in the dynamic and static groups respectively (Figure 6C). Individual analysis of the CD45^+^F4/80^+^DAPI^+^ (Supplementary Figure 9A) and CD45^+^CD68^+^DAPI^+^ (Supplementary Figure 9B) populations as a percentage of the total CD45^+^DAPI^+^ population within the implants confirmed that a high percentage (>90%) of the CD45^+^ population expressed these macrophage markers at 1 and 4 weeks. However, a similar significant decline in the relative expression levels of both individual markers was observed at 8 weeks.

### Dynamic Culture of the Human ASCs on the DAT Scaffolds Prior to Implantation Modulated Macrophage Phenotype Within the Implants in the *nu/nu* Mouse Model

**FIGURE 7:**
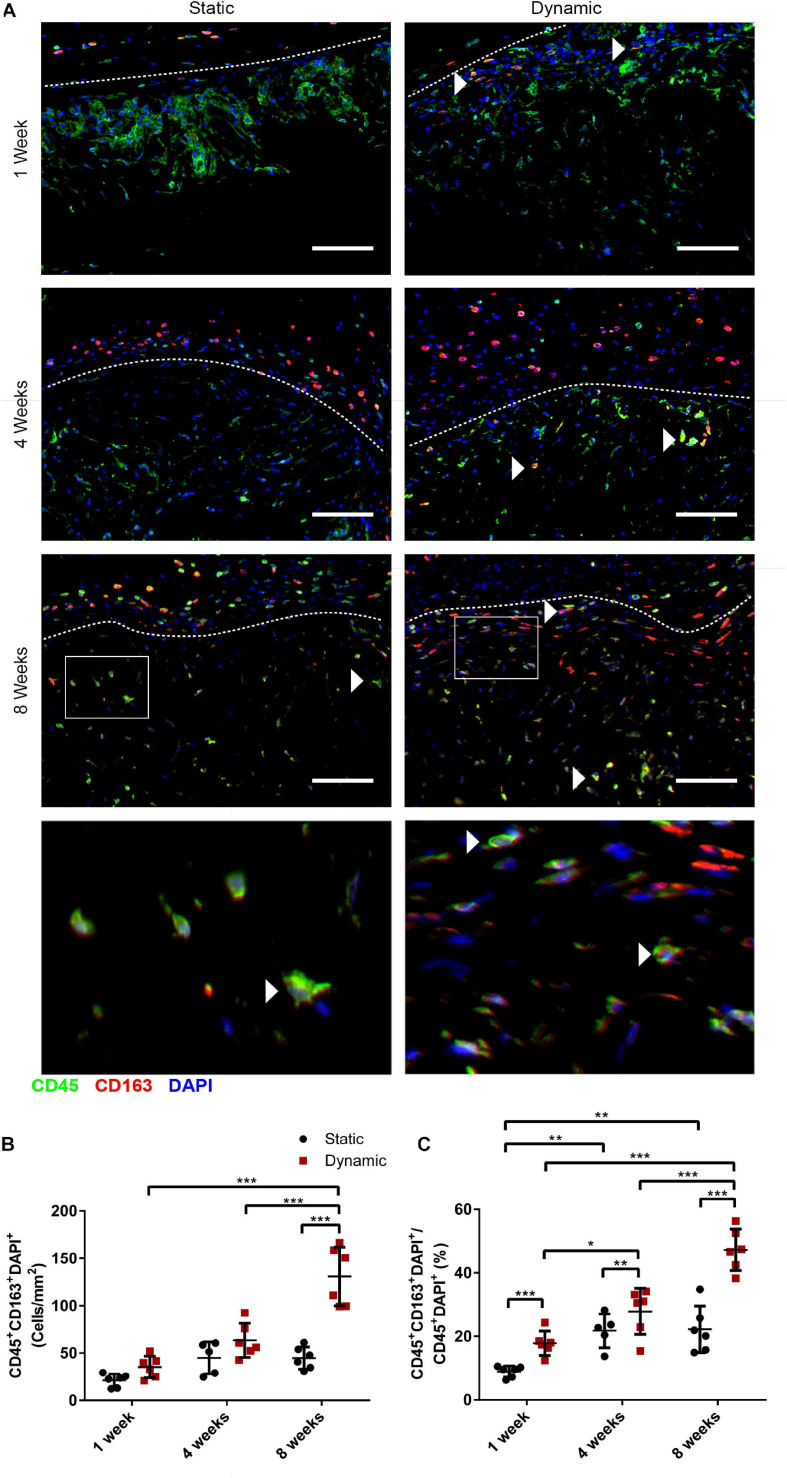
Dynamic culture of human ASCs on the DAT scaffolds enhanced the percentage of cells expressing the pro-regenerative macrophage marker CD163 in the CD45^+^ host cell population at 8 weeks post-implantation in the *nu/nu* mouse model. **(A)** Representative images showing CD45 (green), CD163 (red), and DAPI (blue). Dashed lines indicate the border between the fibrous capsule **(top)** and the DAT implant **(bottom)**. Scale bars represent 100 μm. Boxed regions in the 8-week images are shown at higher magnification below. White arrowheads highlight CD45^+^CD163^+^ cells. **(B)** CD45^+^CD163^+^DAPI^+^ cell density within the DAT implants. **(C)** The percentage of CD45^+^CD163^+^DAPI^+^ cells relative to the total CD45^+^DAPI^+^ population. *p < 0.05, **p < 0.01, ***p < 0.001.

To probe the macrophage phenotype within the implants, co-staining was performed for the pan-leukocyte marker CD45 with the pro-regenerative macrophage marker CD163 (Figure 7A). Interestingly, while CD45^+^ cells were observed infiltrating the implants at 1, 4, and 8 weeks, a relatively small number of CD45^+^CD163^+^ cells were visualized within the scaffolds, with the exception of the dynamic group at 8 weeks. The density of CD45^+^CD163^+^DAPI^+^ cells was quantified within the implanted scaffolds, excluding the fibrous capsule. The analysis confirmed that there were significantly more CD45^+^CD163^+^DAPI^+^ cells within the DAT implants in the dynamic group at 8 weeks (131 ± 31 cells/mm^2^) relative to the static group at 8 weeks (45 ± 12 cells/mm^2^), as well as the dynamic group at 1 (35 ± 11 cells/mm^2^) and 4 (63 ± 18 cells/mm^2^) weeks post-implantation (Figure 7B). When expressed as a percentage of the total CD45^+^DAPI^+^ cell population, a significantly higher fraction of the CD45^+^ cells were CD163^+^ in the dynamic group as compared to the static group at 1 (17.8 ± 3.8% versus 8.9 ± 1.8%), 4 (27.9 ± 7.3% versus 21.8 ± 5.3%) and 8 (47.3 ± 6.5% versus 22.2 ± 7.4%) weeks, with significantly higher levels observed for the dynamic group at 8-weeks post-implantation as compared to both earlier timepoints (Figure 7C), supporting that bioreactor preconditioning promoted a more pro-regenerative macrophage phenotype within the implants over time.

## Discussion

Culturing human ASCs on DAT scaffolds within the perfusion bioreactor under 2% O_2_ over 14 days was previously shown to promote cell expansion and markedly enhance angiogenesis and host adipogenesis following subcutaneous implantation in the *nu/nu* mouse model (Han and Flynn, 2020). The *in vivo* effects were likely attributed in part to the delivery of a significantly larger dose of ASCs in the bioreactor cultured scaffolds relative to the statically cultured controls. However, there is evidence to support that culturing under shear stress can induce functional changes in MSC populations that may influence their capacity to stimulate regeneration (Bassaneze et al., 2010; Yuan et al., 2013; Becquart et al., 2016). As such, the current study sought to explore whether the phenotype and paracrine function of the ASCs on the DAT were altered by preconditioning the scaffolds within the bioreactor.

Inducible nitric oxide synthase and Arg-1 are enzymes involved in distinct pathways of arginine catabolism that have been commonly employed as functional markers of M1 and M2 macrophage polarization, respectively (Rath et al., 2014). In addition, previous studies have shown that iNOS expression is critical for the pro-regenerative immunomodulatory function of murine MSC populations (Ren et al., 2008; Li et al., 2012; Maria et al., 2018). Interestingly, iNOS expression was significantly enhanced in the human ASCs within the peripheral region of the DAT scaffolds in the dynamic group. Further, there was a significantly higher fraction of ASCs that co-expressed iNOS and Arg-1 in the peripheral region of the dynamic group, suggesting that bioreactor preconditioning modulated the ASC phenotype in the periphery of the DAT. In general, Arg-1 was found to be expressed by a high percentage of the ASCs cultured on the DAT scaffolds, which to the best of our knowledge, has not been explored as an MSC marker. However, a previous study reported that an isolated human CD90^+^CD45^–^ intraperitoneal cell population expressed high levels of Arg-1 and suppressed T-cell proliferation in culture, with the immunomodulatory effects regulated by the L-arginine concentration (Rath et al., 2014). While further investigation into the functional role of Arg-1 in human ASCs is warranted, it is possible that the observed expression may be associated with the known anti-inflammatory capacity of these cells (Ceccarelli et al., 2020).

The immunohistochemical analyses also demonstrated that a significantly higher fraction of the ASCs in the peripheral region of the DAT scaffolds in the dynamic group expressed TNF-α and IL-10, suggesting that bioreactor preconditioning promoted the localized production of these factors, which may have modulated the response of infiltrating host cell populations. Although TNF-α and other pro-inflammatory factors are often regarded negatively for their association with chronic inflammation and disease (Bulló et al., 2003; Maachi et al., 2004), it is becoming increasingly recognized that inflammation plays an important role in stimulating angiogenesis and tissue regeneration (Sainson et al., 2008; Kwon et al., 2013). It is important to note that the staining patterns demonstrating higher numbers of cells expressing the markers within the peripheral regions of the DAT (<200 μm from scaffold edge) suggest that media perfusion into the interior of the scaffolds was likely restricted over time within the bioreactor, which we also reported in our previous study (Han and Flynn, 2020). Despite this limitation, the perfusion bioreactor approach was effective at markedly enhancing *in vivo* adipose tissue regeneration within the DAT. While having a more homogeneous population of “activated” ASCs throughout the scaffold could potentially further augment regeneration, it is also possible that the locally enhanced expression of factors such as TNF-α was sufficient to initiate a regenerative response but not shift the overall microenvironment toward a more pro-inflammatory state that may not be conducive for regeneration.

To further assess whether culturing within the bioreactor modulated the paracrine profile of the ASCs on the DAT, a multiplex Luminex assay was performed to characterize the overall expression levels of a range of immunomodulatory factors expressed within adipose tissue. IL-10 expression, which has been shown to promote a more pro-regenerative macrophage phenotype (Mocellin et al., 2003; Boehler et al., 2014) and downregulate the expression of pro-inflammatory cytokines in immune cell populations (Peranteau et al., 2008; Martínez-Chaæon et al., 2018), was significantly enhanced in the dynamic group. In addition, the macrophage chemoattractant CXCL-10 (Petrovic-Djergovic et al., 2015), as well as the pro-angiogenic and anti-inflammatory factor HGF (Beilmann et al., 2004; Ceccarelli et al., 2020), were also detected at significantly higher levels in the lysates from the dynamic group. HGF can regulate endothelial cell survival, proliferation and migration (Kawaguchi and Kataoka, 2014), as well as endothelial tube formation (Beilmann et al., 2004). As such, the enhanced expression of this factor may have played a role in the increased CD31^+^ cell recruitment observed in the *in vivo* model. Further, HGF has also been shown to have immunomodulatory effects on monocytes and macrophages, promoting a more pro-regenerative response (Chen et al., 2014; Choi et al., 2019). The expression of IL-6, which is known to promote the pro-inflammatory polarization of monocytes (Mori et al., 2011; Scheller et al., 2014), was significantly downregulated in the dynamic group. However, no significant differences were observed in the overall protein expression levels of the pro-inflammatory factors TNF-α and CXCL-2 within the lysates between the static and dynamic groups, supporting that the enhanced TNF-α expression observed in the IHC studies was highly localized.

It is possible that shear stress stimulation induced by media perfusion within the bioreactor may have altered the ASC phenotype and secretory profile. Shear forces can activate mechanosignaling pathways, including the MAPK and JNK pathways, through integrin activation, tensegrity-mediated mechanosensing, and by inducing changes in the fluidity of the cell membrane (Stolberg and McCloskey, 2009). Human ASCs have also been shown to possess a primary cilium (Hilgendorf et al., 2019), which has the potential to function as a shear stress sensor and signal transducer. Most studies to date exploring biomechanical stimulation on MSCs have focused on differentiation, rather than on possible effects on pro-angiogenic or immunomodulatory capacities. However, mechanical stimulation through intermittent fluid flow has previously been reported to increase the production of a range of pro-angiogenic and immunomodulatory factors including VEGFA, HGF, G-CSF, and IL-8 in cultured human ASCs (Bravo et al., 2017). Similarly, Becquart et al. (2016) demonstrated that intermittent shear stress stimulated the production of nitric oxide (NO) and enhanced mRNA expression of *VEGFA*, *FGF2*, and *IGF1* in cultured human bone marrow-derived MSCs. In addition, Lee et al. (2017) recently demonstrated that human bone marrow-derived MSCs cultured under physiological shear stress within a microfluidic system showed enhanced anti-inflammatory function, with the effects regulated through focal adhesion kinase (FAK) signaling.

Notably, in addition to shear stress other factors could have contributed to the altered protein expression levels observed between the groups in the current study, including differences in the cell density, variations in the local concentration of nutrients, oxygen or other metabolites, as well as a cascading effect of other growth factors or cytokines. For example, in lipopolysaccharide (LPS)-induced macrophages *in vitro* and in interstitial macrophages in C57BL/6J mice, the enhanced expression of HGF was found to decrease IL-6 and increase IL-10 expression (Kamimoto et al., 2009). Further studies using alternative bioreactor platforms would be required to more specifically assess the effects of fluid shear stress on the pro-angiogenic and immunomodulatory functionality of the ASCs.

Building from the *in vitro* data, *in vivo* studies were performed to assess the effects of bioreactor preconditioning on host cell recruitment. Consistent with our previous findings that there was a significant increase in the density of erythrocyte-containing blood vessels in the DAT implants in the dynamic group at 4 and 8 weeks (Han and Flynn, 2020), CD31^+^ endothelial cell recruitment was significantly enhanced in the dynamic group at all three timepoints in the current study, suggesting that culturing within the bioreactor augmented the capacity of the ASCs to stimulate angiogenesis within the implants. As it is well-recognized that the induction of angiogenesis is required for stable adipose tissue formation within engineered bioscaffolds (Ceccarelli et al., 2020), this may have contributed to the increased adipogenesis observed within this group (Han and Flynn, 2020).

The infiltration of host-derived CD26^+^ cells was probed as a marker that has been associated with an adipocyte precursor phenotype. More specifically, Merrick et al. (2019) demonstrated that a CD26^+^ interstitial progenitor population within murine adipose tissue could give rise to an intermediate population that was committed to the adipogenic lineage and could differentiate into mature adipocytes *in vivo*. The higher density of host-derived CD26^+^ cells observed at 1 week post-implantation in the dynamic group, combined with the previously characterized adipo-inductive effects of this group (Han and Flynn, 2020), suggests that the dynamically cultured DAT scaffolds have the potential to both recruit progenitors and drive their differentiation toward an adipogenic state, associated with downregulation of CD26 expression (Merrick et al., 2019). However, it should be noted that other cell types can express CD26, including dendritic cells (Gliddon and Howard, 2002; Zhong et al., 2013), macrophages (Zhong et al., 2013), and natural killer cells (Bühling et al., 1994), and future studies should more fully characterize the CD26^+^ population and its involvement in adipogenesis within the DAT.

Macrophages have been indicated to play an important role in mediating both angiogenesis and adipose tissue regeneration within ECM-derived bioscaffolds (Debels et al., 2013; Han et al., 2015). As such, the effects of dynamic culture on host macrophage recruitment were probed through co-staining for CD45, F4/80, and CD68, which showed no significant differences in terms of cell densities between the groups at any of the timepoints. Similarly, we previously found that seeding with ASCs modulated macrophage phenotype but did not specifically alter macrophage recruitment into the DAT implants in immunocompetent rat and mouse models (Han et al., 2015; Robb et al., 2020). Notably, there was an increased percentage of CD45^+^ cells that were F4/80^–^ and CD68^–^ within both implant groups at 8 weeks. These cells may be other myeloid cell populations or their precursors, or potentially T cells (Kennedy et al., 1992) or natural killer cells (Krzywinska et al., 2016). It would be interesting to more fully characterize these cells to better understand their potential effects within the implants in future studies, including additional markers with quantitative analysis of digested explants using multicolor flow cytometry.

While there was no difference in total macrophage recruitment, analysis of CD163 expression in the CD45^+^ cell population suggested that dynamic preconditioning of the ASCs on the DAT ultimately led to a shift toward a more pro-regenerative macrophage phenotype. In particular, CD163^+^ macrophages have been associated with both angiogenesis and ECM remodeling in implanted biomaterials (Spiller et al., 2014). The increased presence of CD163^+^ macrophages within the dynamically cultured implants is similar to our previous findings on the effects of ASC seeding in the immunocompetent rat and mouse models (Han et al., 2015; Robb et al., 2020), and these cells may have contributed to the enhanced remodeling of the DAT into host-derived adipose tissue (Han and Flynn, 2020).

Based on the *in vitro* and *in vivo* data in the current study, we postulate that the markedly enhanced adipose tissue regeneration observed following implantation of bioreactor preconditioned DAT scaffolds was due to the combined effects of (i) the delivery of a larger number of cells and (ii) alterations in the ASC phenotype and paracrine function within the periphery of the DAT. Addressing limitations in the current study, future work should focus on decoupling these effects and verifying that the ASCs that expressed the markers such as iNOS did indeed have enhanced pro-angiogenic and/or immunomodulatory capacities. These studies should include functional assays, for example, probing specific effects on endothelial cell proliferation and tubule formation, as well as macrophage polarization. For follow-up *in vivo* studies, especially those focused on characterizing the immunomodulatory effects of the ASCs, testing in humanized mouse models should be considered (Mehler et al., 2019). Using these models, it would be interesting to more fully characterize the phenotype of the infiltrating macrophages over time, and assess the role of other immune cell populations including T-cells. It would also be beneficial to perform studies using human ASCs engineered to stably express luciferase to enable more accurate tracking of the viable cells within the living animals through longitudinal bioluminescence imaging.

Although preconditioning the cells within the 3-D perfusion bioreactor system on the same scaffold on which they were delivered was effective within our pre-clinical model, it may be worthwhile to consider separate platforms for cell preconditioning versus cell delivery as a potentially more scalable approach to advance toward future clinical translation as a cell-based therapy for large volume soft tissue augmentation. More specifically, alternative bioreactor systems could be designed that combine shear stress stimulation with culturing on ECM-derived bioscaffolds where the cells could be more uniformly subjected to the applied forces to avoid the heterogeneity that was observed in the cellular response in the current study. For example, our previously established stirred bioreactor system for expanding the ASCs on DAT microcarriers could hold promise as a dynamic preconditioning platform (Yu et al., 2017). The preconditioned cells could subsequently be incorporated within other biomaterial scaffolds designed to provide structural support and maintain the volume as host adipose tissue regeneration progresses, such as the intact DAT scaffolds used in the current study.

## Conclusion

The findings of the current study support that dynamic preconditioning of human ASCs on the DAT scaffolds within the perfusion bioreactor under 2% O_2_ altered their phenotype and paracrine profile relative to controls cultured under static conditions. Interestingly, a substantial fraction of the ASCs within the peripheral region of the DAT implants in the dynamic group simultaneously expressed iNOS and Arg-1, as well as both TNF-α and IL-10, suggesting they had a complex phenotype. Analysis in the *in vivo* model indicated that culturing within the bioreactor prior to implantation enhanced the recruitment of CD31^+^ endothelial cells, as well as CD26^+^ host cells, into the implants, which likely contributed to the increased angiogenesis and adipogenesis previously reported in this group. While there was no significant difference in total macrophage recruitment between the groups, greater infiltration of CD163^+^ macrophages was observed in the dynamic group, indicative of a shift toward a more pro-regenerative macrophage response favorable for implant remodeling. Taken together with our previous work, the *in vitro* and *in vivo* findings suggest that bioreactor preconditioning can augment the capacity of human ASCs to stimulate adipose tissue regeneration within the DAT, and that these effects are likely mediated by a combination of increased expansion resulting in the delivery of a larger number of ASCs, as well as alterations in the paracrine functionality of the delivered ASCs. Overall, the current study supports the further investigation of dynamic culture under shear stress as a means to precondition ASCs and enhance their capacity to stimulate tissue regeneration through paracrine-mediated mechanisms.

## Data Availability Statement

The raw data supporting the conclusions of this article will be made available by the authors, without undue reservation.

## Ethics Statement

The studies involving human participants were reviewed and approved by the Health Sciences Research Ethics Board, Western University. The patients/participants provided their written informed consent to participate in this study. The animal study was reviewed and approved by the Animal Care Committee, Western University.

## Author Contributions

LF and TH conceptualized and designed the study, with input from GD on macrophage characterization and AG on translational perspectives. AG provided clinical tissue samples for cell isolation and scaffold fabrication. TH performed the experimental studies and analyzed the data in collaboration with JW and LF, and in consultation with GD. TH and LF wrote the manuscript, with editorial feedback provided by JW, GD, and AG. All authors contributed to the article and approved the submitted version.

## Conflict of Interest

The authors declare that the research was conducted in the absence of any commercial or financial relationships that could be construed as a potential conflict of interest.
