# Supplementary material for: Preconditioning Human Adipose-Derived Stromal Cells on Decellularized Adipose Tissue Scaffolds Within a Perfusion Bioreactor Modulates Cell Phenotype and Promotes a Pro-regenerative Host Response
Source: Front Bioeng Biotechnol. 2021 Mar 18;9:642465. doi: 10.3389/fbioe.2021.642465 (PMC8012684; doi:10.3389/fbioe.2021.642465)
Supplement: Supplementary file 1 [file Data_Sheet_1.docx]

Supplementary Material


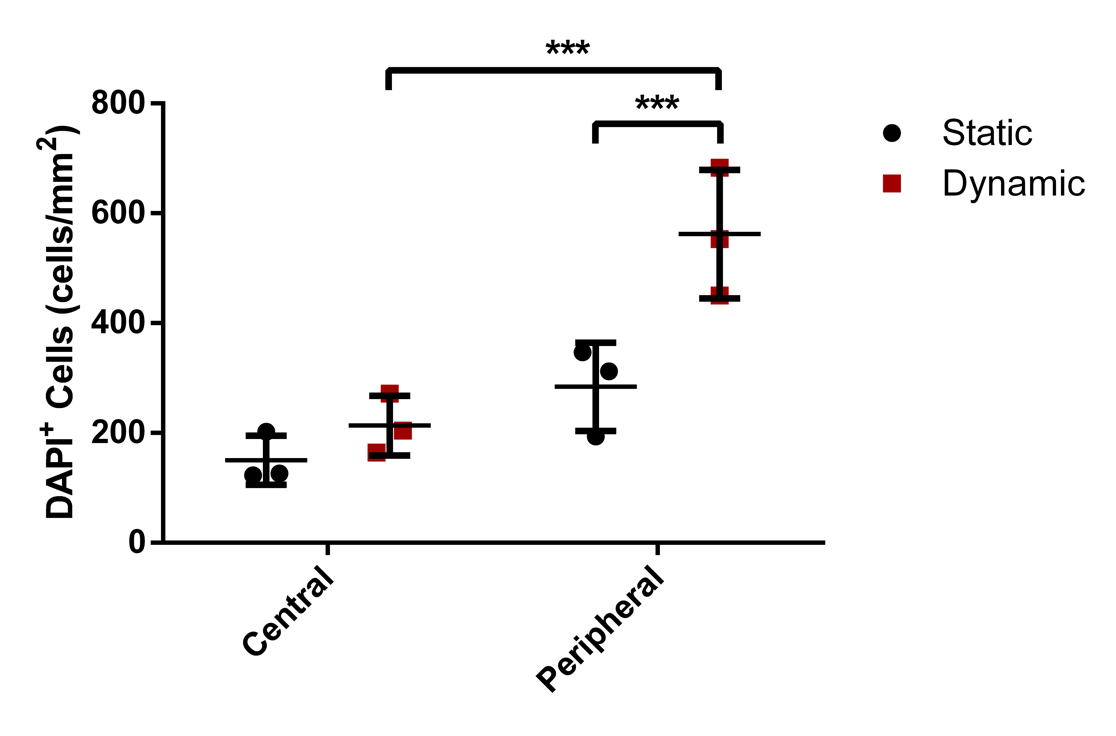


**Supplementary Figure 1.** DAPI quantification confirmed that the ASC density was significantly higher in the peripheral region as compared to the central region of the DAT scaffolds that had been cultured within the bioreactor for 14 days, as well as compared to the peripheral region of the scaffolds that had been cultured statically for 14 days. *** p<0.001.


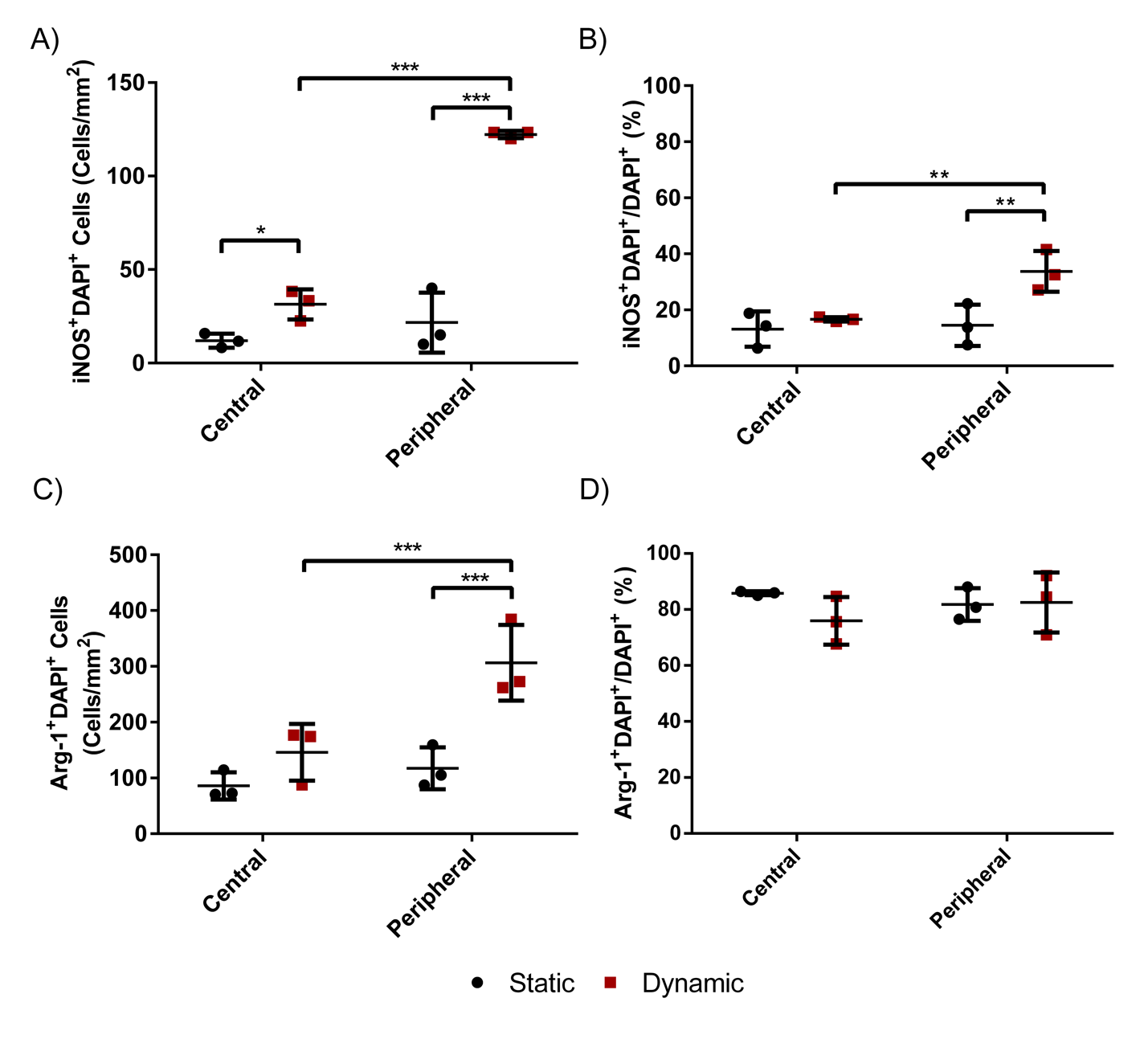


**Supplementary Figure 2.** Analysis of iNOS and Arg-1 expression in the ASC-seeded DAT scaffolds cultured *in vitro* statically or dynamically for 14 days. A) The density of iNOS^+^DAPI^+^ cells and B) iNOS^+^DAPI^+^ cells as a percentage of the total DAPI^+^ cell population, and C) the density of Arg‑1^+^DAPI^+^ cells and D) Arg-1^+^DAPI^+^ cells as a percentage of the total DAPI^+^ cell population in both the central (> 200 µm from scaffold border) and peripheral (< 200 µm from scaffold border) regions of the DAT scaffolds. * p<0.05, ** p<0.01, *** p<0.001.


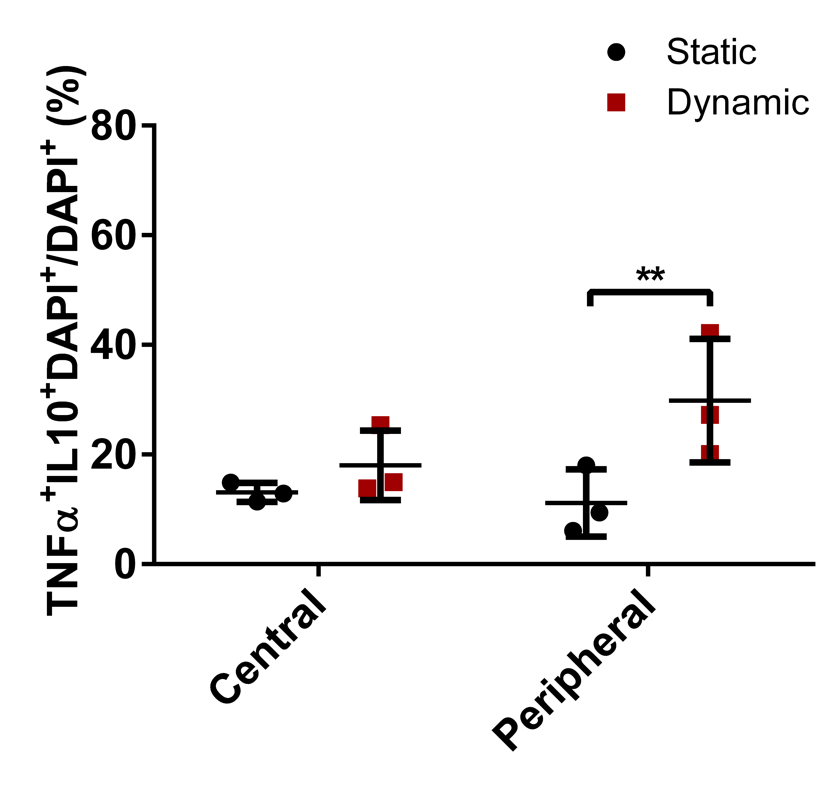


**Supplementary Figure 3.** The percentage of the total DAPI^+^ human ASC population that was TNF‑α^+^IL-10^+^DAPI^+^ in the central (> 200 µm from scaffold border) and peripheral (< 200 µm from scaffold border) regions of the DAT scaffolds that had been cultured statically or dynamically for 14 days. ** p<0.01.

**
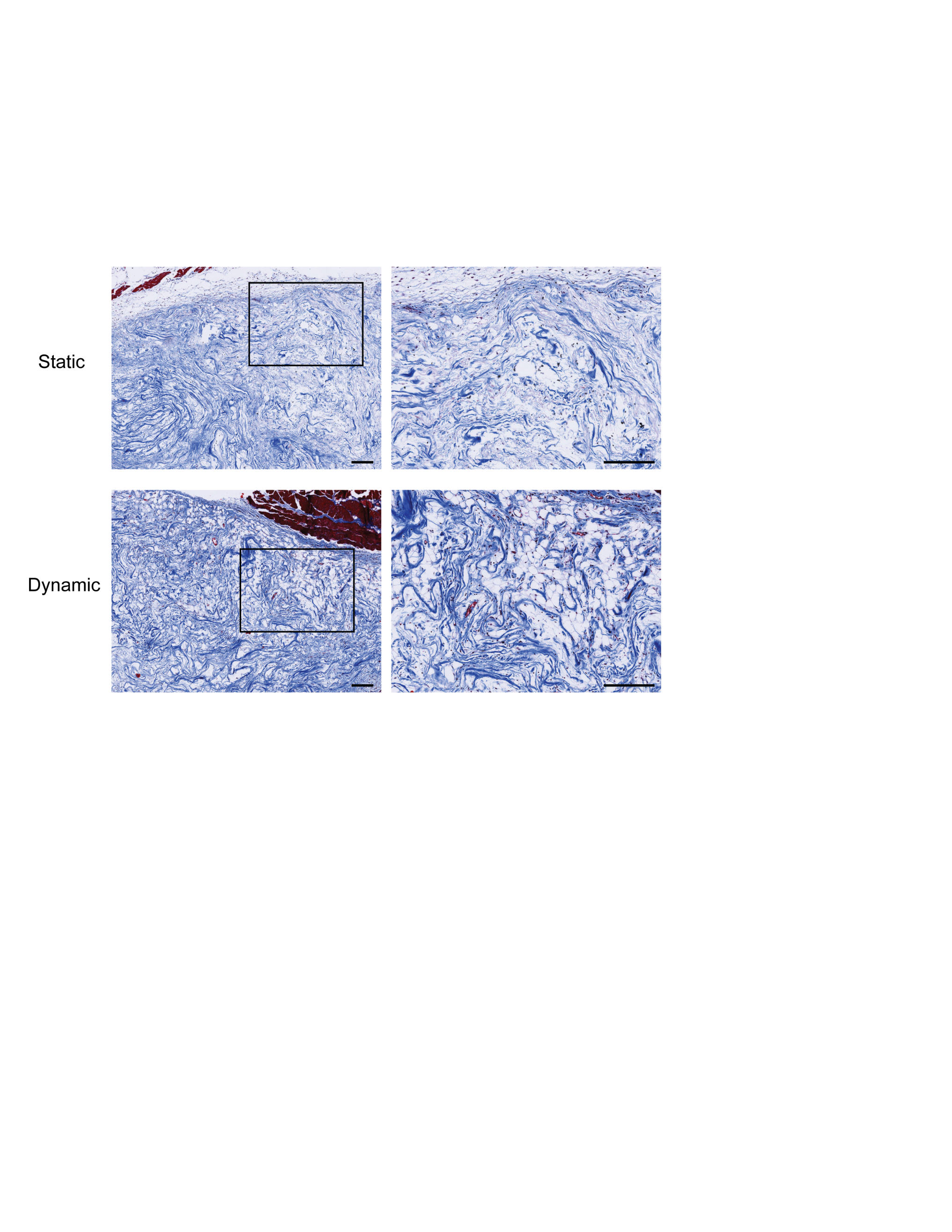
**

**Supplementary Figure 4.** Representative Masson’s trichrome staining showing enhanced remodeling of the human ASC-seeded DAT implants at 8 weeks post-implantation in the scaffolds that were cultured for 14 days within the perfusion bioreactor prior to implantation in athymic nude mice as compared to static cultured controls. Boxed regions are shown at higher magnification on right, demonstrating the increased presence of adipocytes and blood vessels within the implant region in the dynamic culture group. Scale bars represent 200 µm.

**
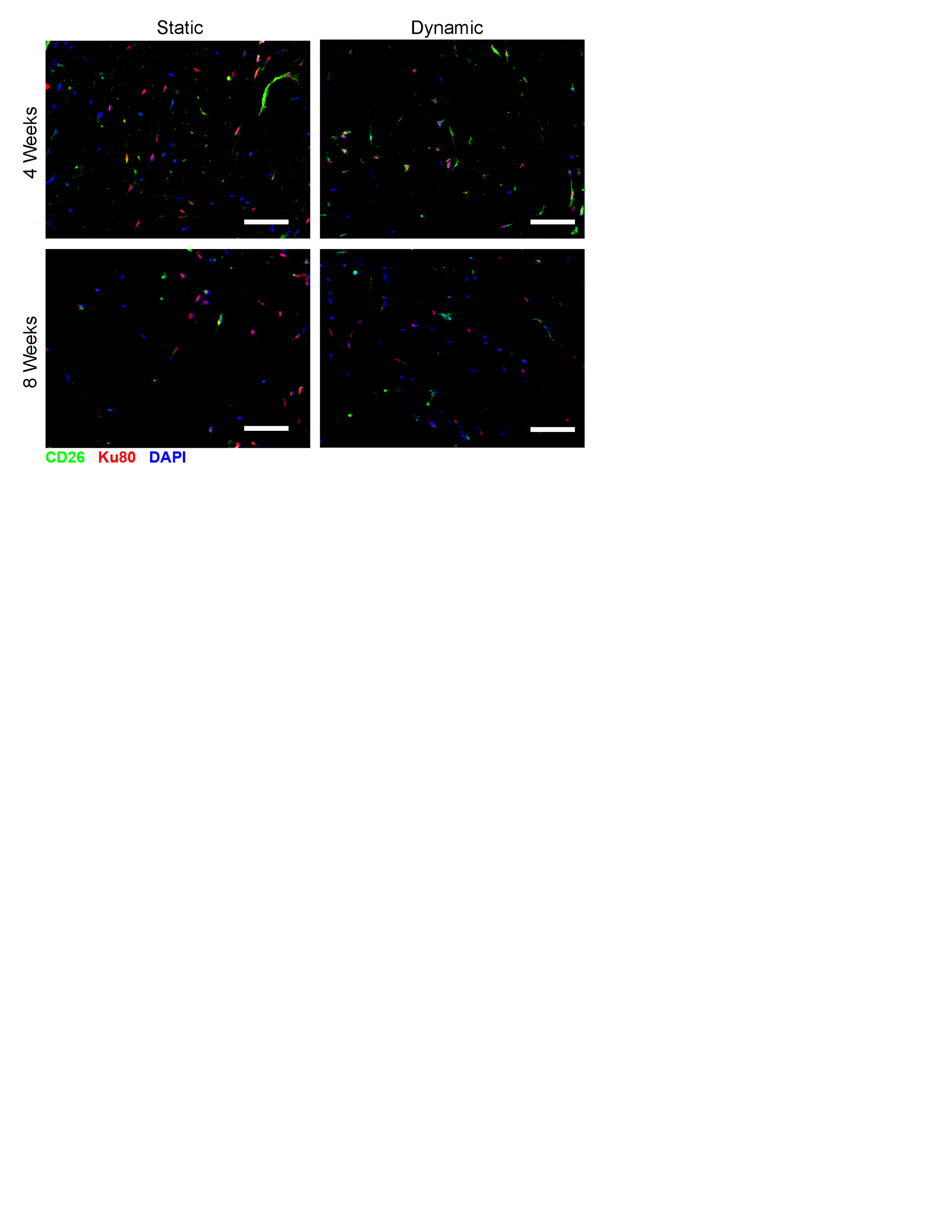

Supplementary Figure 5.** Representative immunostaining for CD26 (green) and Ku80 (red) with DAPI counterstaining (blue) in the static and dynamic implants at 4 and 8 weeks. Scale bars represent 100 µm.

**
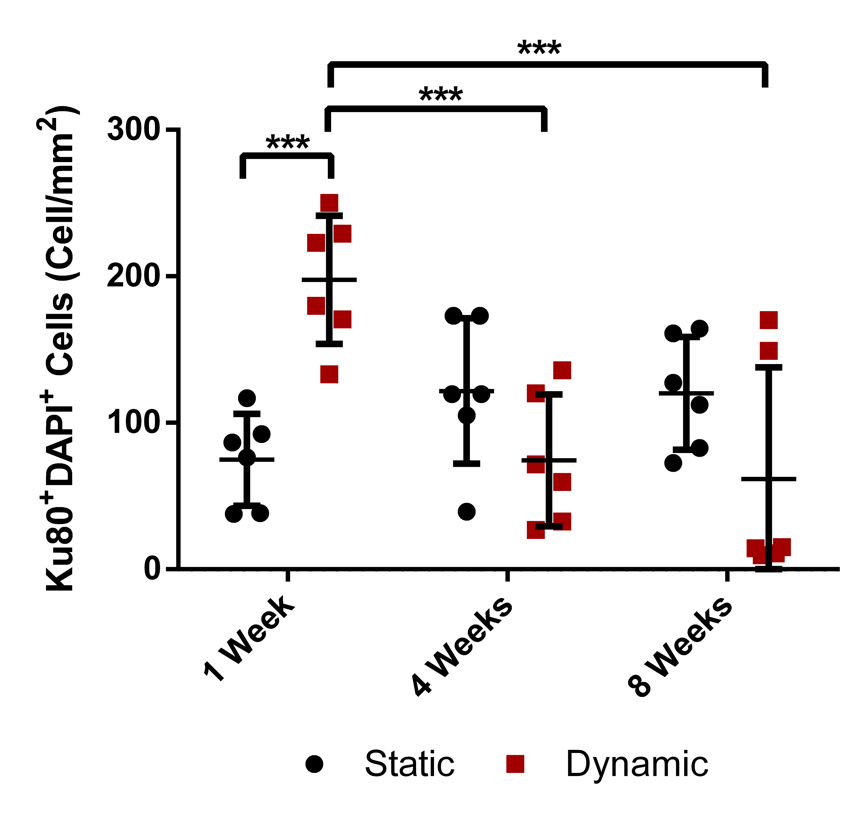
**

**Supplementary Figure 6.** The density of Ku80^+^DAPI^+^ human ASCs within the DAT implants at 1, 4 and 8 weeks. *** p<0.001.


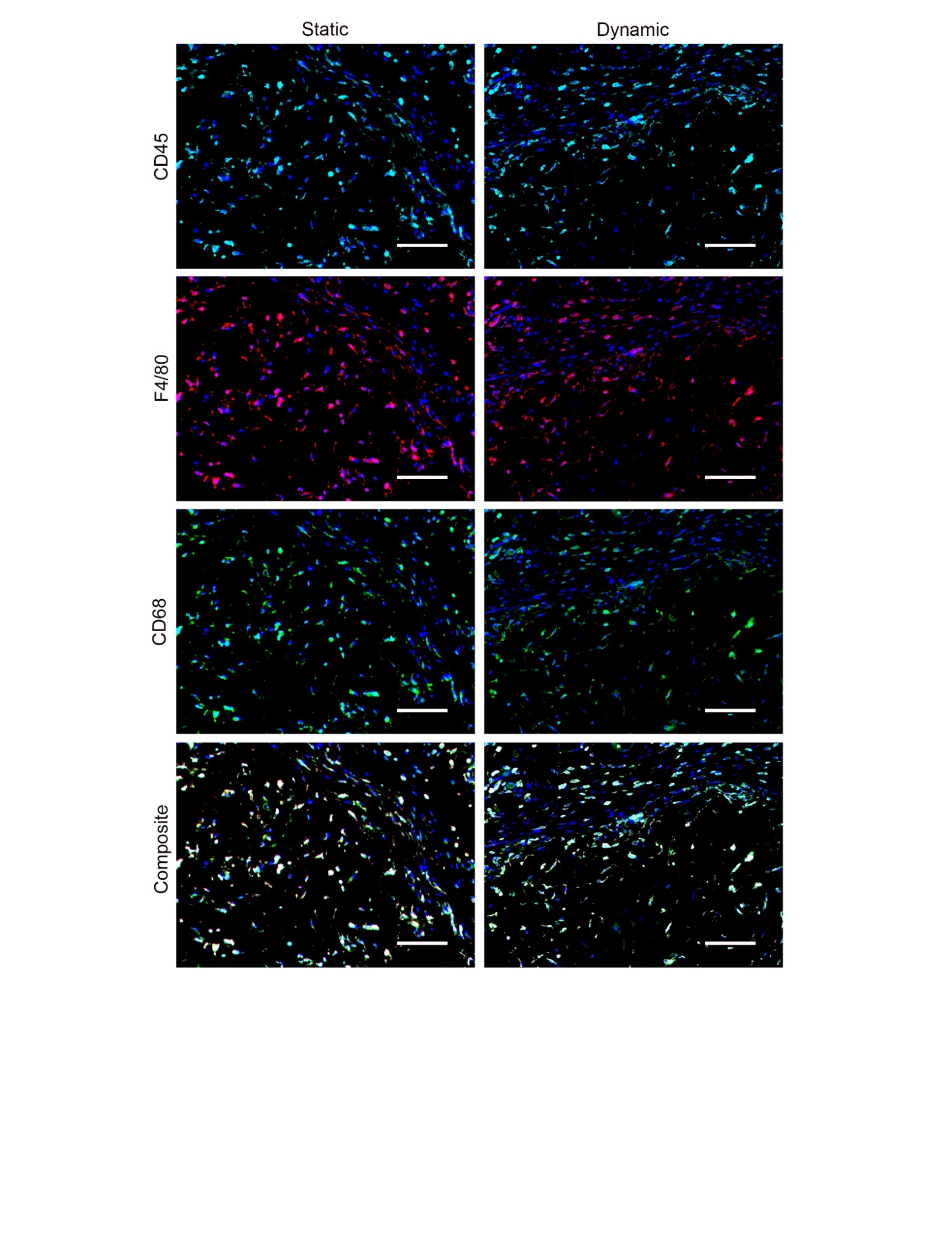


**Supplementary Figure 7.** Representative immunostaining for CD45 (cyan), F4/80 (red) and CD68 (blue) with DAPI counterstaining (blue) in the static and dynamic implants at 4 weeks. Scale bars represent 100 µm.


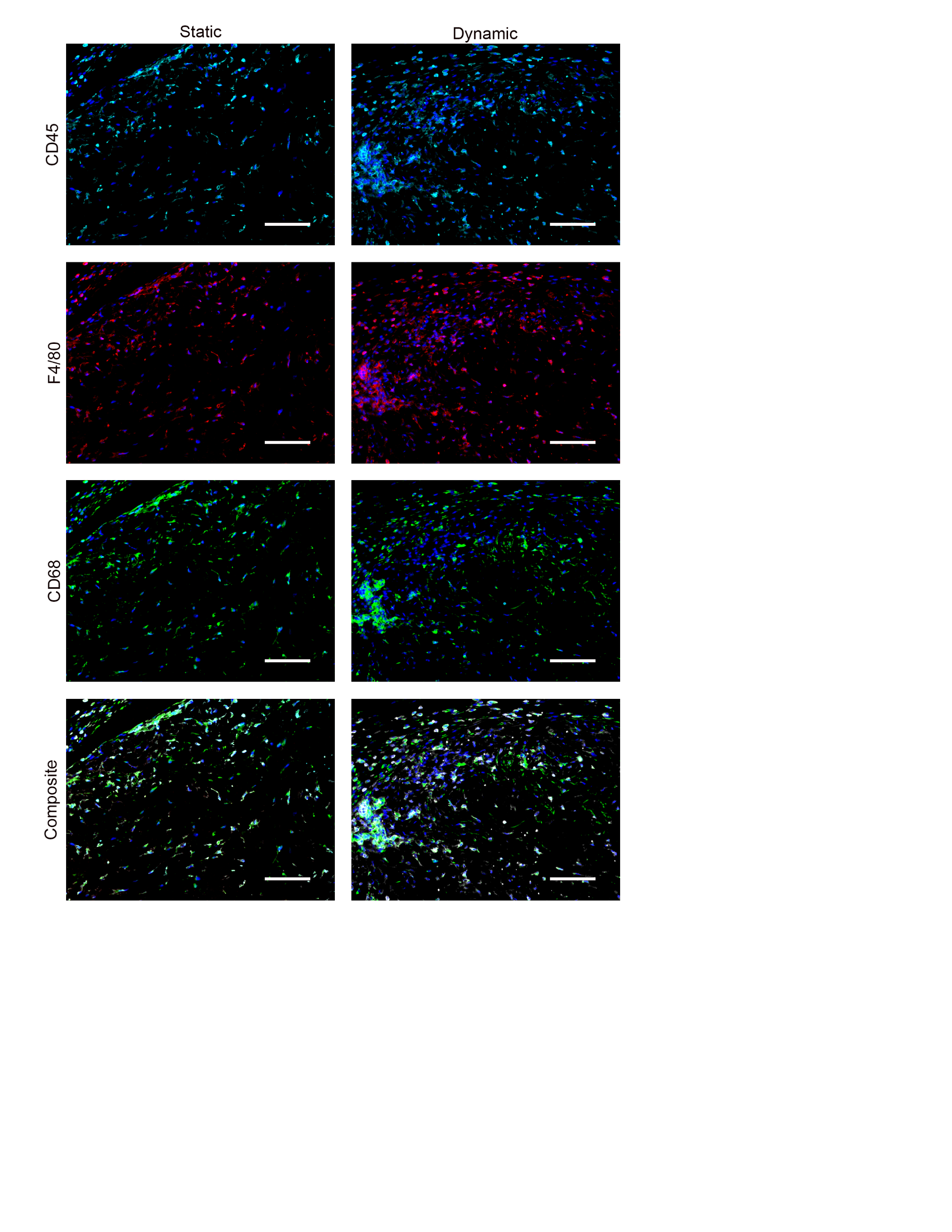


**Supplementary Figure 8.** Representative immunostaining for CD45 (cyan), F4/80 (red) and CD68 (blue) with DAPI counterstaining (blue) in the static and dynamic implants at 8 weeks. Scale bars represent 100 µm.

**
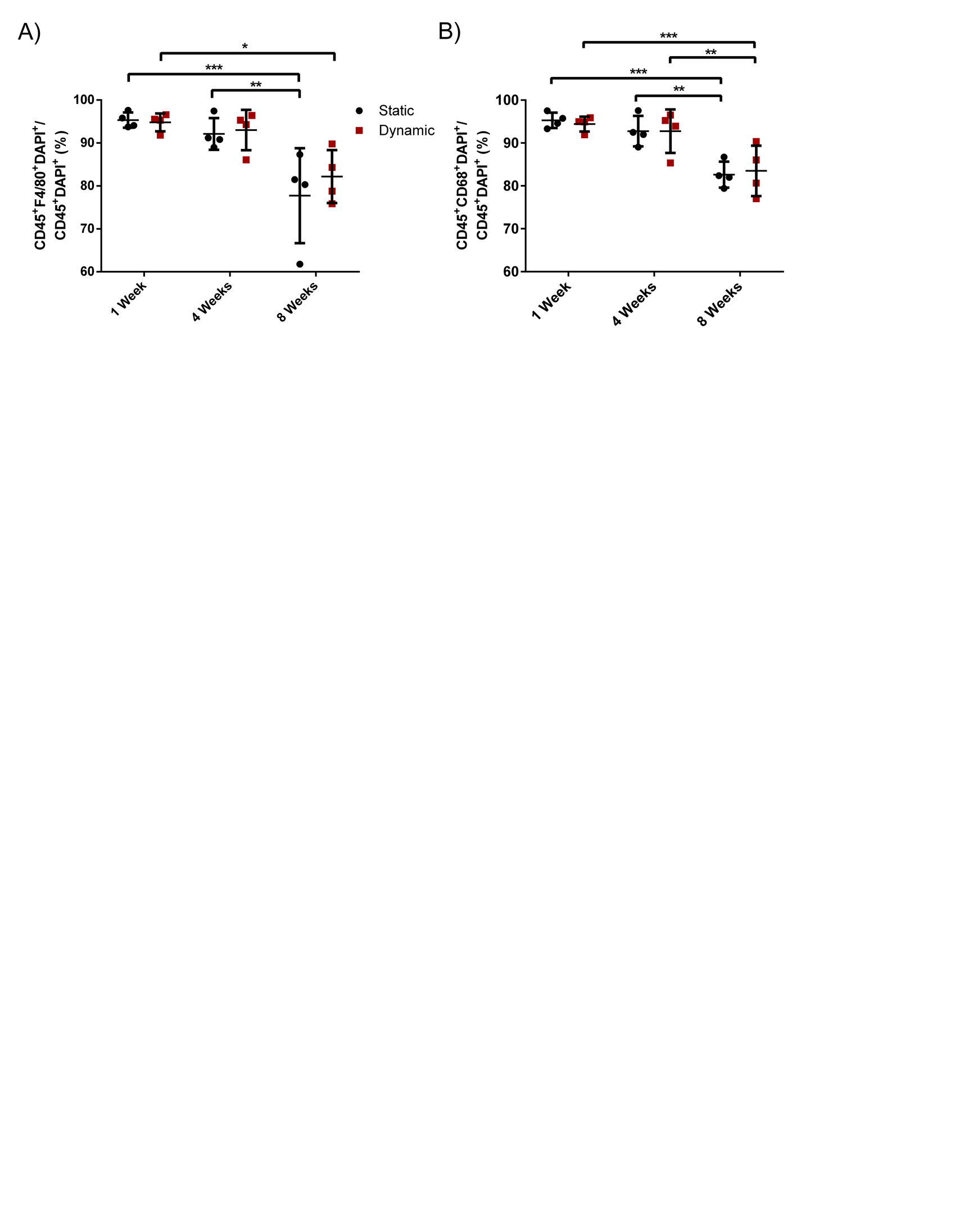
**

**Supplementary Figure 9.** The percentage of A) CD45^+^F4/80^+^DAPI^+^ cells and B) CD45^+^CD68^+^DAPI^+^ relative to the total CD45^+^DAPI^+^ cell population in the DAT implants at 1, 4 and 8 weeks. * p<0.05, ** p<0.01, *** p<0.001.
